# Commentary: Effect of high-dose *Spirulina* supplementation on hospitalized adults with COVID-19: a randomized controlled trial

**DOI:** 10.3389/fimmu.2024.1417046

**Published:** 2024-08-29

**Authors:** Elias E. Mazokopakis, Maria G. Papadomanolaki

**Affiliations:** ^1^ Department of Internal Medicine, Naval Hospital of Crete, Chania, Greece; ^2^ School of Production Engineering and Management, Technical University of Crete, Chania, Crete, Greece

**Keywords:** *Spirulina*, *Arthrospira platensis*, supplementation, COVID-19, prevention, hospitalization

## Introduction

Spirulina is a filamentous cyanobacterium known for its high nutritional value and therapeutic properties. There is growing evidence that Spirulina (*Arthrospira platensis)* supplementation can contribute to the war against SARS-CoV2, either preventing COVID-19 and reducing the need for hospitalization (1) or reducing mortality in hospitalized patients with COVID-19 (2).

## Study of interest

The interesting study by Mohammad Reza Aghasadeghi et al. (2) about the beneficial effect of high-dose Spirulina supplementation on hospitalized adults with COVID-19. A report by the authors is not entirely correct and needs to be corrected.

## Discussion

The authors in *Discussion* section report that their study represents the first published report of a clinical trial examining high-dose Spirulina platensis as a dietary supplement in hospitalized COVID-19 patients, such as the majority of previous investigations have been centered on animal and *in vitro* studies (2). With full respect to the authors, this point of view is not entirely correct, because they ignored our published study in the year 2022 which investigated the role of Spirulina supplementation on COVID-19 prevention and hospitalization (1). This 6-month study included 186 (median age: 47, range: 30-60 years) healthy Greek individuals, non-vaccinated against the COVID-19. Among the 102 unvaccinated individuals who received orally 6 g high quality Spirulina (*Arthrospira platensis;* produced by the Hellenic Spirulina Net, Thermopigi, Sidorokastro, Greece) daily for 6 months, only 14 (13.7%) contracted SARS-CoV2 (confirmed Delta variant) with mild symptoms and 2 (1.9%) needed hospitalization because of acute viral gastroenteritis. In contrast, among the 84 unvaccinated individuals who did not receive Spirulina, 62 (73.8%) contracted SARS-CoV2 (confirmed Delta variant) with mild symptoms and 17 (20.2%) needed hospitalization. None of the 19 hospitalized patients with COVID-19 received Spirulina supplement in the hospital. Also, none of the 19 hospitalized patients died. Our study revealed that Spirulina supplementation at a dose of 6 g daily can contribute to the war against SARS-CoV2, preventing COVID-19 and reducing the need for hospitalization. In the past we have also published studies on the hepatoprotective and hypolipidemic effects of Spirulina (3, 4).

## References

[1] Mazokopakis EE Papadomanolaki MG. The contribution of Spirulina platensis supplementation on COVID-19 prevention and hospitalization. EJMED. (2022) 4:82–3. doi: 10.24018/ejmed.2022.4.3.1355

[2] Aghasadeghi MR Zaheri Birgani MA Jamalimoghadamsiyahkali S Hosamirudsari H Moradi A Jafari-Sabet M. Effect of high-dose Spirulina supplementation on hospitalized adults with COVID-19: a randomized controlled trial. Front Immunol. (2024) 15:1332425. doi: 10.3389/fimmu.2024.1332425 38655258 PMC11036872

[3] Mazokopakis EΕ Starakis IK Papadomanolaki MG Mavroeidi NG Ganotakis ES. The hypolipidemic effects of *Spirulina* (Arthrospira platensis) supplementation in a Cretan population: a prospective study. J Sci Food Agric. (2014) 94:432–7. doi: 10.1002/jsfa.6261 23754631

[4] Mazokopakis EE Papadomanolaki MG Fousteris AA Kotsiris DA Lampadakis IM Ganotakis ES. The hepatoprotective and hypolipidemic effects of Spirulina *(Arthrospira platensis)* supplementation in a Cretan population with non-alcoholic fatty liver disease (NAFLD): a prospective pilot study. Ann Gastroenterol. (2014) 27:387–94. PMC4188938 25331487

